# Real-time on-site detection of the three ‘*Candidatus* Liberibacter’ species associated with HLB disease: a rapid and validated method

**DOI:** 10.3389/fpls.2023.1176513

**Published:** 2023-06-07

**Authors:** Félix Morán, Mario Herrero-Cervera, Sofía Carvajal-Rojas, Ester Marco-Noales

**Affiliations:** ^1^ Instituto Valenciano de Investigaciones Agrarias (IVIA), Centro de Protección Vegetal y Biotecnología, Unidad de Bacteriología, Moncada, Valencia, Spain; ^2^ Universidad de Costa Rica (UCR), Centro de Investigación en Biología Celular y Molecular (CIBCM), Laboratorio de Fitopatógenos Obligados y sus Vectores (LaFOV), San José, Costa Rica

**Keywords:** Huanglongbing, rapid-screening test, in situ, KIT, RPA (recombinase polymerase amplification)

## Abstract

Huanglongbing (HLB) is a devastating disease that affects all commercial citrus species worldwide. The disease is associated with bacteria of three species of the genus ‘*Candidatus* Liberibacter’ transmitted by psyllid vectors. To date, HLB has no cure, so preventing its introduction into HLB-free areas is the best strategy to control its spread. For that, the use of accurate, sensitive, specific, and reliable detection methods is critical for good integrated management of this serious disease. This study presents a new real-time recombinase polymerase amplification (RPA) protocol able to detect the three ‘*Ca*. Liberibacter’ species associated with HLB in both plant and insect samples, validated according to European and Mediterranean Plant Protection Organization (EPPO) guidelines and tested on 365 samples from nine different geographic origins. This new protocol does not require nucleic acid purification or specialized equipment, making it ideal to be used under field conditions. It is based on specific primers and probe targeting a region of *fusA* gene, which shows a specificity of 94%–100%, both *in silico* and *in vitro*, for the ‘*Ca*. Liberibacter’ species associated with HLB. The analytical sensitivity of the new protocol is excellent, with a reliable detection limit in the order of 10^1^ copies per microliter in HLB-infected plant and insect material. The repeatability and reproducibility of the new methods showed consistent results. Diagnostic parameters of the new RPA protocol were calculated and compared with the gold standard technique, a quantitative real-time PCR, in both crude extracts of citrus plants and insect vectors. The agreement between the two techniques was almost perfect according to the estimated Cohen’s kappa index, with a diagnostic sensitivity and specificity of 83.89% and 100%, respectively, and a relative accuracy of 91.59%. Moreover, the results are obtained in less than 35 min. All these results indicate the potential of this new RPA protocol to be implemented as a reliable on-site detection kit for HLB due to its simplicity, speed, and portability.

## 1 Introduction

Citrus is the most widely grown fruit crop in the world, so threats to this crop pose a risk to the socio-economic network of many countries. One of the weak points of citrus is its vulnerability to fungi, bacteria, and viruses (Ghosh et al., 2022), which can alter the normal growth of the plant, the development of leaves and branches, and the organoleptic properties of the fruit.

To date, one of the most destructive diseases for citrus is the Huanglongbing (HLB), also known as citrus greening, which affects all commercial citrus species and cultivars, severely reducing both crop yield and tree longevity (Bové, 2006).

HLB disease is associated with three uncultured bacterial species of the genus *Liberibacter* (family Rhizobiaceae, class Alphaproteobacteria, order Rhizobiales): ‘*Candidatus* Liberibacter asiaticus’ (CaLas), ‘*Candidatus* Liberibacter africanus’ (CaLaf), and ‘*Candidatus* Liberibacter americanus’ (CaLam). These bacteria live in the plant phloem and are mainly transmitted by sap-sucking insects of the family Psyllidae. Specifically, the Asian citrus psyllid *Diaphorina citri* (Hemiptera: Liviidae) is the natural vector of CaLas and CaLam, and the African citrus psyllid *Trioza erytreae* (Hemiptera: Triozidae) is the natural vector of CaLaf, although, under experimental conditions, the African psyllid is capable to transmit CaLas (Massonie et al., 1976; Reynaud et al., 2022), and the Asian citrus psyllid is capable of transmitting CaLaf (Lallemand et al., 1986).

The three ‘*Ca*. Liberibacter’ species associated with HLB are spread across several countries in Asia, Africa, and the Americas (EPPO Global Database, 2023), where they are causing devastation to millions of citrus trees in major citrus-producing countries (Das, 2008; Das et al., 2014; Neupane et al., 2016; da Costa et al., 2021). At present, none of these three bacteria have been reported in the Mediterranean Basin or Australia. However, in recent years, as a result of globalization, climate change, and the presence of the two insect vectors in the Mediterranean Basin (Pérez Otero et al., 2015; Wang, 2020; Ministry of Agriculture and Rural Development of Israel, 2022), HLB disease has become the most serious threat to the citrus sector in this area, especially for important European citrus- exporting countries, such as Spain.

Typical symptoms of HLB include diffuse yellow mottling of leaves, reduction in fruit size, and loss of organoleptic quality, as well as stunting of trees (Bové, 2006; Dala-Paula et al., 2019). As bacteria move slowly along vessels with a heterogeneous distribution throughout the different parts of the tree (Tatineni et al., 2008), symptoms appear several years after the first infection, making detection a challenge. Moreover, the asymptomatic spread of the disease hinders its control (Lee et al., 2015).

At present, the HLB citrus disease has no cure, and the best way to prevent its spread is to implement integrated disease management, applying a combination of strategies that include the early detection of bacteria in order to eradicate infected trees (Alquezar et al., 2022). In this context, it is essential the development of efficient, accurate, and rapid HLB diagnostic tools for specific bacterial detection.

Over the years, many different techniques have been developed for HLB diagnosis (Valdés et al., 2016; Ghosh et al., 2022), but molecular techniques are currently the most widely used for the diagnosis of plant pathogenic bacteria, including those associated with HLB, as they have a higher sensitivity and specificity than others (Sapre et al., 2021). Within the molecular detection techniques, the polymerase chain reaction (PCR) is the gold standard reference technique for HLB diagnosis (Valdés et al., 2016; Morán et al., 2020). In this regard, according to the latest HLB diagnostic protocols published by the European and Mediterranean Plant Protection Organization (EPPO) and the International Plant Protection Convention (IPPC), PCR protocols that amplify partial regions of several genes (Jagoueix et al., 1996; Hocquellet et al., 1999; Teixeira et al., 2005; Li et al., 2006; Lin et al., 2010; Morgan et al., 2012; Zheng et al., 2016; de Chaves et al., 2023) are the most recommended for the detection of ‘*Ca*. Liberibacter’ spp. in both symptomatic and asymptomatic plant samples and insect vectors (EPPO, 2021b; IPPC-FAO, 2022).

Although PCR-based detection methods are indeed the most appropriate for laboratory screening, they have several limitations when used outside of a laboratory setting (Ivanov et al., 2021), as they require specialized equipment, trained personnel, and, in most cases, a nucleic acid purification step, making them not the most suitable tool for a first quick *in situ* diagnosis. Therefore, it is necessary to look for alternative techniques that involve less sample processing time and simple equipment for rapid on-site diagnosis.

In the last decade, new techniques based on isothermal amplification of nucleic acids have emerged, and their use has increased in the diagnosis of plant pathogenic microorganisms (Patel et al., 2022). They are a good option for rapid field detection. In recent years, several HLB detection protocols based on isothermal amplification have been developed: eight LAMP protocols (Li et al., 2012; Rigano et al., 2014; Ghosh et al., 2016; Wu et al., 2016; Qian et al., 2017; Choi et al., 2018; Stolowicz et al., 2022; Thoraneenitiyan et al., 2022) and three recombinase polymerase amplification (RPA) protocols (Ghosh et al., 2018; Li et al., 2022; Rattner et al., 2022). All these isothermal amplification methods were designed to detect a single species of ‘*Ca*. Liberibacter’ associated with HLB, CaLas, probably because it is the most widespread species, is the most related to huge economic losses, and has the highest potential for spread (Lee et al., 2015; Taylor et al., 2019; Ajene et al., 2020). However, climate change and the consequences of accelerated globalization could increase the possibility of other HLB-associated bacteria invading disease-free areas as well. Thus, for HLB-free regions, such as the Mediterranean Basin and Australia, CaLam and CaLaf also pose a serious hazard that should not be underestimated, even more so when the African vector, *T. erytreae*, is already present and expanding in Spain and Portugal. In addition, it is very important that a method of such significance be validated according to the standards of organizations in the scope of agriculture and plant protection, which guarantees its robustness and reliability.

Therefore, the objective of this work has been the development and validation, according to EPPO guidelines, of a rapid, sensitive, and specific detection method for the diagnosis of HLB, which can be applicable in on-site conditions. For this purpose, we have used real-time RPA methodology, selecting highly specific primers and probe for the three species of ‘*Ca*. Liberibacter’ associated with this devastating citrus disease.

## 2 Material and methods

### 2.1 Plant and insect material

A total of 141 plant and insect samples and bacterial suspensions were quantified by real-time PCR (de Chaves et al., 2023) and comparatively analyzed by the new real-time RPA developed in this work (**Supplementary Table S1**). Particularly, 131 samples of nine different ‘*Ca*. Liberibacter’ spp. hosts from nine different geographic origins were used for the evaluation of the analytical specificity of the new detection method.

More precisely, a total of 76 positive samples used for validation included the following: i) symptomatic and asymptomatic plant material from species *Citrus sinensis* cv. Valencia, *Citrus hystrix*, *Citrus* sp., and Rusk citrange from Brazil (São Paulo and Paraná), Costa Rica (Alajuela), Cuba (Artemisa), and the plant collection of National Research Institute for Agriculture Food and the Environment (INRAE); and ii) insect samples of the species *D. citri* from the USA. A total of 65 negative samples included plant material of the species *C. sinensis* cv. Lane Late, *Citrus clementina* cv. Clemenules, and *Citrus limon* cv. Fino Mesero from Spain (Valencia) and insect samples of *D. citri* from the USA (Florida) and *T. erytreae* from Spain and South Africa. In addition, four suspensions at 10^6^ CFU/ml of bacterial species *Agrobacterium tumefaciens* (strain C58/ATCC 33970), *Agrobacterium vitis* (strain 339), *Liberibacter crescens* (strain BT-1), ‘*Ca*. Liberibacter solanacearum’, *Xylella fastidiosa* subsp. *pauca* (CFBP 8072), *X. fastidiosa* subsp. *fastidiosa* (IVIA 5770), *X. citri* subsp. *citri* (CFBP 2911), *Spiroplasma citri* (NCPPB 3095), and *Pseudomonas syringae* pv. *syringae* (IVIA 2827) were used as bacterial negative controls.

In order to evaluate the new detection method under field conditions, a total of 226 samples were collected in three random surveys performed in two countries where HLB disease is present and one country where the disease is absent: 140 samples were collected in Brazil (Matão, São Paulo), 66 in Costa Rica (Santa Eulalia, Alajuela), and 20 in Spain (Moncada, Valencia) (geographical coordinates, DD, according to Google Maps: −21.53, −48.6; 10.02, −84.36; and 39.58, −0.39 respectively). Surveys were conducted on private land, with permission of the owners, and in accordance with current national and international legislation, as no special permits were required.

The presence/absence and absolute quantification of ‘*Ca*. Liberibacter spp.’ in all samples were determined using the real-time qPCR detection protocol described by de Chaves et al. (2023) from DNA purification of each sample. The identification of the ‘*Ca*. Liberibacter’ species associated with HLB was performed by partial Sanger sequencing of the 16S rRNA gene following the protocol described by Morán et al. (2020).

### 2.2 Crude extract preparation and DNA purification

Plant samples were prepared from five citrus leaf petioles, which were placed and ground in individual plastic bags (Bioreba, Reinach, Switzerland) containing phosphate-buffered saline (PBS) (8.0 g of NaCl, 2.9 g of Na_2_HPO_4_·12 H_2_O, 0.2 g of KH_2_PO_4_, and 0.2 g of KCl; pH 7.2) at a rate of 1:5 (w:v). Insect samples were prepared from individual specimens, which were squashed on Whatman paper (GE Healthcare, Europe), according to the method described by Bertolini et al. (2014); the membranes were then resuspended with 100 µl of 0.05% Tween 20.

Fresh crude plant extracts and insect extracts were used as direct samples for RPA analysis and to perform DNA purifications. DNA was purified from 400 µl of crude plant extracts and 90 µl of squashed insect extracts following the cetyl trimethylammonium bromide (CTAB) method recommended by the EPPO protocol PM7/121 (2) (EPPO, 2021b), without the addition of beta-mercaptoethanol. Quantification of total DNA was performed using a DeNovix DS-11 spectrophotometer (DeNovix Inc., Wilmington, DE, USA). Crude plant and squashed insect extracts and DNA purifications were stored at −20°C until use.

### 2.3 Complete genome analysis and design of primers and probe for recombinase polymerase amplification

The search for a highly conserved region was performed by multiple alignments of 11 complete genomes (accessed January 2023) using the package MAUVE (Darling et al., 2004). Specifically, nine complete genomes of ‘*Ca*. Liberibacter asiaticus’ (GenBank accession numbers: NZ_CP010804.2, NC_020549.1, NZ_AP014595.1, NZ_CP019958.1, NC_012985.3, NZ_CP061535.1, NZ_CP040636.1, NZ_CP054558.1, and NZ_CP041385.1) (Zhou et al., 2011; Lin et al., 2013; Katoh et al., 2014; Zheng et al., 2014; Petrone et al., 2019; Lu et al., 2021; Wang et al., 2021; Zheng et al., 2022a; Zheng et al., 2022b), one of ‘*Ca*. Liberibacter africanus’ (NZ_CP004021.1) (Lin et al., 2015), and one of ‘*Ca*. Liberibacter americanus’(NC_022793.1) (Wulff et al., 2014) were aligned. To select the most appropriate RPA target region, the level of conservation of 17 housekeeping genes (*rplL*, *rplJ*, *rpoB*, *rpoC*, *recA*, *glnA*, *fumC*, *gyrA*, *gyrB*, *metG*, *fusA*, *infB*, *mutS*, *grpE*, *dnaA*, *dnaG*, and *atpD*) was evaluated based on their alignment identity percentage (AIP) values.

The Proksee tool (Proksee, n.d.) was used to visualize the genomic map by BLAST comparison of the different isolates of ‘*Ca*. Liberibacter’ spp.

The design of primers and probe of RPA was accomplished following the recommendations stipulated in the TwistAmp^®^ Assay Design Manual (TwistAmp®, 2018). The free online tool OligoAnalyzer™ 3.1 (Owczarzy et al., 2008) (OligoAnalyzer Tool - IDT) was used to check the GC content percentage and melting temperature and to identify the secondary structures of the primers and probe.

Primers and probe of the new real-time RPA were designed in a region of *fusA* gene targeting a sequence of 201 bp. Primers designed were RPA-HLB-F2 (5′-ATAAAARTCCGC[deoxyinosine]CCCATCTTATCCATTTTATTG-3′) and RPA-HLB-R2 (5´-TATTGATACTCCTGG[deoxyinosine]CARGTTGATTTTACTA-3´), and the probe for detection of the amplification product was RPA-HLB-P2 (5′-ATACTTATCAGCCTGACGCCATACCGTTTC [FAM-dT][THF][BHQ1-dT]TTGCGGTTCAACACC[Spacer3]-3′). To detect the three ‘*Ca*. Liberibacter’ spp. associated with HLB, deoxyinosine molecules were included in both primers as internal modifications.

### 2.4 Primer test by conventional PCR and basic RPA

To evaluate the new primers designed for RPA, they were tested in conventional PCR and RPA formats. Conventional PCR was performed using GoTaq^®^ Hot Start Polymerase (Promega Corporation, USA). The reaction mixture contained 1× GoTaq^®^ Hot Start Polymerase PCR buffer, 3 mM of MgCl_2_, 0.2 mM of each dNTP, 600 mM of each RPA primer, and 2.5 U of GoTaq^®^ DNA polymerase in a total volume of 25 μl with 50 ng of DNA template. PCR amplification was performed in a Veriti 96 Well thermal cycler (Applied Biosystems, Foster City, CA, USA), and conditions consisted of one initial denaturalization cycle of 3 min at 94°C followed by 40 cycles of 30 s at 94°C, 40 s at 50°C, and 45 s at 72°C, with a final extension step of 72°C for 10 min.

Basic RPA was performed using TwistAmp Liquid Basic kit (TwistDx, Maidenhead, UK) following the manufacturer’s indications. Optimized reaction mix consisted of 1× reaction buffer, 0.5 mM of each dNTP, 1× Basic E-mix, 1× Core Reaction Mix, 600 mM of each RPA primer, and 19 mM of MgOAc in a total volume of 50 µl with 1 µl of crude extract. RPA amplification conditions were adjusted in a Veriti 96 Well thermal cycler (Applied Biosystems, Foster City, CA, USA), consisting of a single incubation of 30 min at 45°C. All mixtures were shaken 5 min after the start of the reaction.

Amplicons were purified using mi-PCR Purification Kit (Metabion International AG, Germany) and visualized in 1.5% (w/v) agarose gel with GoodView™ staining (SBS Genetech Co., Ltd., China) after electrophoresis in 0.5× TAE buffer (40 mM of Tris; 20 mM of CH_3_COOH; 1 mM of EDTA, pH 7.6).

### 2.5 Real-time RPA

For real-time RPA, the TwistAmp Liquid Exo kit (TwistDx, Maidenhead, UK) was used according to the manufacturer’s instructions. The optimized reaction mix consisted of 1× reaction buffer, 0.45 mM of each dNTP, 1× Probe E-mix, 600 mM of each RPA primer, 200 nM of RPA probe, 1× Core Reaction Mix, 1× Exo, and 19 nM of MgOAc in a total volume of 50 µl with 1 µl of crude extract. Reaction incubation and fluorescence signal reading were performed using fluorimeters AmplifyRP^®^ XRT (Agdia, Île-de-France, France) or Genie^®^ II (OptiGene, Horsham, UK), consisting of 45°C for 35 min. All mixtures were shaken 5 min after the start of the reaction.

### 2.6 Validation of real-time RPA

The real-time RPA method developed in this study was validated according to the guidelines described in the EPPO diagnostic protocol for regulated pests PM 7/98 (5) (EPPO, 2021a) about the specific requirements for laboratories preparing accreditation for a plant pest diagnostic activity.

Thus, the following parameters were evaluated: i) analytical sensitivity, ii) analytical specificity, iii) selectivity, iv) repeatability, v) reproducibility, vi) diagnostic sensitivity and specificity, and vii) relative accuracy.

#### 2.6.1 Analytical sensitivity

Analytical sensitivity of real-time RPA was evaluated by testing, in triplicate, 10-fold serial dilutions of healthy extracts of *C. sinensis* cv. Lane spiked with known amounts (10^5^ to 4 copies/µl) of synthetic gBlocks (Integrated DNA Technologies, USA) containing the RPA target sequence (223 bp) of *fusA* gene from species CaLas, CaLaf, and CaLam (NZ_CP019958.1; NZ_CP004021.1 and NC_022793.1) (**Supplementary Material**). Avogadro constant was used to estimate the number of double- stranded DNA (dsDNA) copies (6.023 × 10^23^ molecules/mol) in each dilution.

Analytical sensitivity was also evaluated in plant and insect material by two approaches: one using HLB-infected material diluted in healthy extracts and the other using crude healthy extracts spiked with synthetic dsDNA. For HLB-infected material, three replicates of 10-fold serially diluted (from 10^−1^ to 10^−6^) crude CaLas-infected extracts (samples 12,112.6 and 11,661.7) in healthy extracts were analyzed. Absolute quantification of CaLas was performed in each dilution by real-time PCR according to de Chaves et al. (2023).

Furthermore, in order to evaluate the limit of detection (LOD) in infected samples, 76 positive samples infected with CaLas, CaLaf, and CaLam isolates were quantified by real-time qPCR (de Chaves et al., 2023), and the absolute result was compared with the new real-time RPA protocol.

#### 2.6.2 Analytical specificity and selectivity

Inclusivity was evaluated by analyzing a range of different isolates of ‘*Ca*. Liberibacter’ species from Brazil, Cuba, Costa Rica, INRAE collection, and the USA, infecting *C. sinensis* cv. Valencia, *Citrus × hystrix*, *Citrus × reshni*, Rusk citrange, *Citrus* sp., and *D. citri*. A total of 35 amplicons positive by real-time RPA, which were selected as representative of different origins and hosts, were purified as described above and sequenced through Sanger sequencing methods in both directions (forward and reverse DNA strands) using the new RPA primers designed (see Section 3.1).

Sequences were analyzed using Geneious Prime 2022 software (Biomatters Ltd., Auckland, New Zealand) and submitted to GenBank database. In addition, *in silico* inclusivity and exclusivity were evaluated by BLASTN comparison of the designed RPA primers and probe against nucleotide NCBI database of RefSeq genomes of Rhizobiaceae family (NCBI:txid82115 accessed January 2023).

The exclusivity of real-time RPA was assessed by analyzing a set of relevant non-target bacteria composed of species phylogenetically close to those associated with HLB (*L. crescens* and ‘*Ca*. Liberibacter solanacearum’) and citrus pathogenic species (*P. syringae* pv. *syringae*, *X. fastidiosa*, *X. citri* subsp. *Citri*, and *S. citri*) (**Supplementary Table S1**).

Real-time RPA selectivity was evaluated by testing extracts of HLB-infected samples from four different citrus cultivars and one vector species from five different geographical origins (**Supplementary Table S1**). In addition, symptomatology in plant material was taken into account in random surveys performed in countries where HLB is present, in order to test the detection capability of real-time RPA in both symptomatic and asymptomatic plants.

#### 2.6.3 Repeatability and reproducibility

The repeatability and reproducibility of real-time RPA were evaluated using 10 plant samples (473, 607, 614, 615, 623, 627, 631, 633, 635, and 641) of *C. sinensis* cv. Valencia, originating from Costa Rica (Alajuela), infected with relatively low CaLas titers (low Cqs values, according to the real-time qPCR test by de Chaves et al. (2023). Each crude extract sample selected was analyzed using two fluorimeters: AmplifyRP^®^ XRT (Agdia, Île-de-France, France) and Genie^®^ II (OptiGene, Horsham, UK). For each instrument, three independent replicates were carried out on different days and by two different operators.

#### 2.6.4 Diagnostic sensitivity and specificity

Validation of real-time RPA, by comparison with real-time qPCR (de Chaves et al., 2023), was performed on 226 total samples collected in two random surveys conducted in Costa Rica and Brazil, as described in Section 2.1. Each sample was analyzed in triplicate by real-time qPCR and real-time RPA. In order to confirm the correct *fusA* gene amplification by real-time RPA, a selection of 27 positive samples from random surveys were sequenced as described in Section 2.6.2.

Correlation results (PA, positive agreement; PD, positive deviation; ND, negative deviation; NA, negative agreement) were used to calculate the diagnostic sensitivity (PA/[PA + ND]), diagnostic specificity (NA/[NA + PD]), and relative accuracy ([PA + NA]/[PA + PD + ND + NA]) of real-time RPA. The agreement between techniques was evaluated using Cohen’s kappa index (Cohen, 1960), which indicates the proportion of agreement beyond that expected by chance. The benchmarks of Landis and Koch (Landis and Koch, 1977) were used to categorize Cohen’s kappa index, where < 0.00 is poor agreement, 0 to 0.2 is slight agreement, 0.21 to 0.40 is fair agreement, 0.41 to 0.60 is moderate agreement, 0.61 to 0.80 is substantial agreement, and 0.81 to 1.00 is almost perfect agreement.

## 3 Results

### 3.1 Design and evaluation of RPA primers and probe

**Figure 1:**
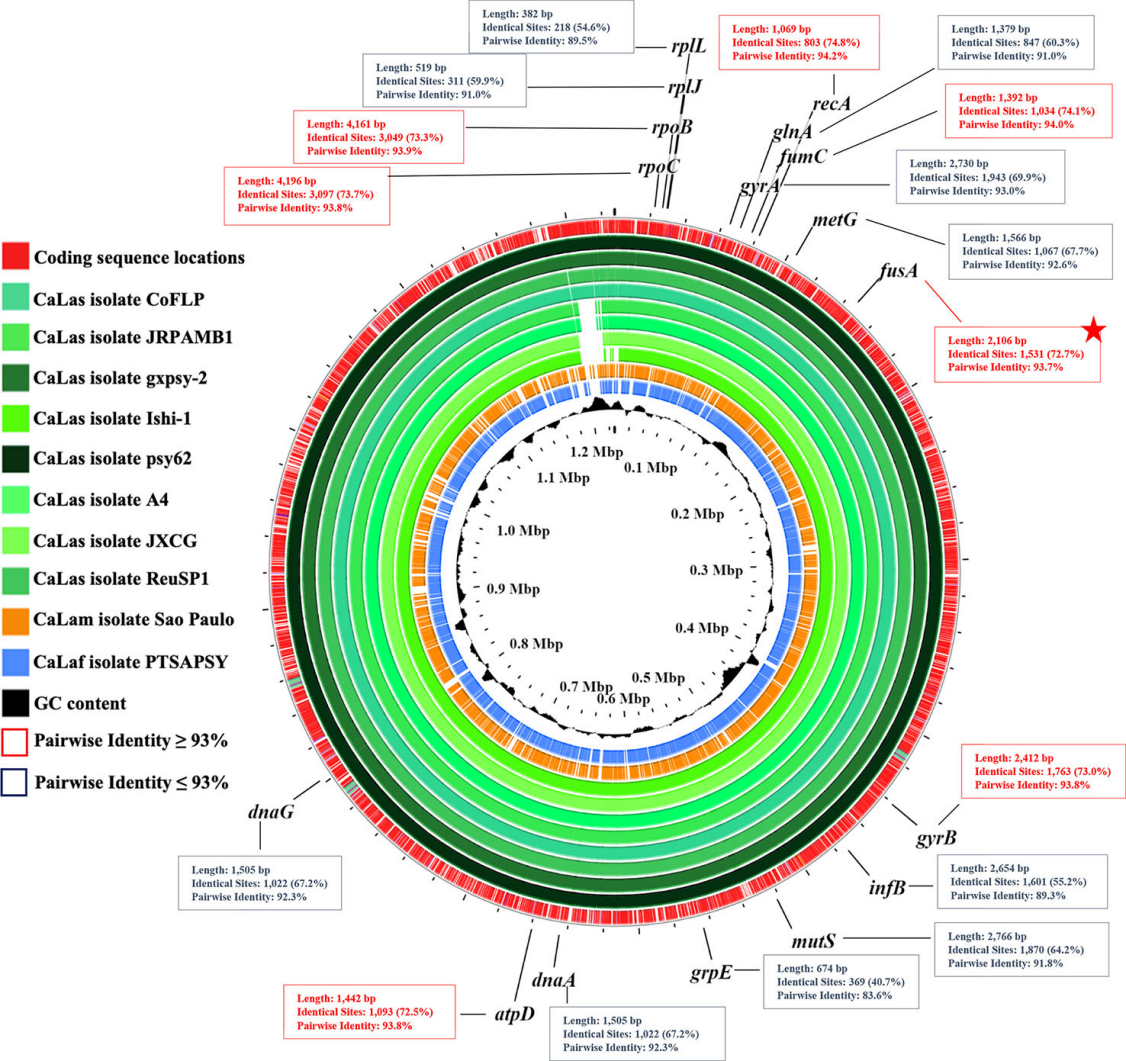
Genomic map, performed by BLASTN with Proksee tool, of 11 ‘*Candidatus* Liberibacter’ isolates associated with Huanglongbing (HLB) disease from different origins (isolates psy62, gxpsy, A4, Ishi-1, JXGC, JRPAMB1, TaiYZ2, CoFLP, ReuSP1, PTSAPSY, and São Paulo). The map shows the 17 housekeeping genes evaluated for the design of primers for RPA technique (*rplL*, *rplJ*, *rpoB*, *rpoC*, *recA*, *glnA*, *fumC*, *gyrA*, *gyrB*, *metG*, *fusA*, *infB*, *mutS*, *grpE*, *dnaA*, *dnaG*, and *atpD*), with their MAUVE alignment pairwise identity percentage values. Genes with pairwise identity values ≥ 93% are highlighted in red boxes, and red star shows the target gene selected for the design of primers and probe.

Complete genome alignment of 11 ‘*Ca*. Liberibacter’ isolates associated with HLB disease (psy62, gxpsy, A4, Ishi-1, JXGC, JRPAMB1, TaiYZ2, CoFLP, ReuSP1, PTSAPSY, and São Paulo) was used to select the most appropriate RPA target region. Genes *rplL*, *rplJ*, *glnA*, *gyrA*, *metG*, *infB*, *mutS*, *grpE*, *dnaA*, and *dnaG* were discarded for having AIP values ≤ 93%. Since high AIP values increase the probability of finding conserved regions that allow the design of oligos capable of hybridizing only with the three *Liberibacter* species associated with HLB, housekeeping genes *rpoB*, *rpoC*, *recA*, *fumC*, *fusA*, *gyrB*, and *atpD*, with AIP values ≥ 93%, were initially selected for primer and probe design (**Figure 1**). Finally, *fusA* gene, with AIP 93.7%, was the one that presented a more appropriate region for the design of the primers and the probe, which are usually large (30–36 nt for primers and 46–52 nt for probe). The gene *fusA*, which is present in a single copy per genome, encodes the elongation factor G, a molecule involved in the protein translation process in prokaryotes.

The results of the *in silico* specificity evaluation of the RPA primers, performed by BLASTN against RefSeq genomes of the Rhizobiaceae family, showed high inclusivity and exclusivity. Percentage identity ranged from 85% to 100% only with ‘*Ca*. Liberibacter’ species, and no nucleotide homologies were found with any other species of the family Rhizobiaceae. Specifically, the primers showed 94%–100% identities with the ‘*Ca*. Liberibacter’ spp. associated with HLB and identity values below 91% with ‘*Ca*. Liberibacter solanacearum’. While *in silico* evaluation of the specificity of the RPA probe showed good inclusivity, with 85%–100% sequence homology with the HLB-associated ‘*Ca*. Liberibacter’ spp. with respect to exclusivity, the RPA probe showed identity homologies lower than 85% with other bacterial species of the Rhizobiaceae family, such as *Ensifer mexicanus*, *A. vitis*, *Rhizobium glycinendophyticum*, and *Rhizobium croatiense*, whose hosts are not citrus plants.

With respect to *in vitro* specificity evaluation of RPA primers, performed by conventional PCR and RPA, amplification of total DNA from 35 positive control samples selected among citrus plant material and insect vectors (**Supplementary Table 1**) showed the expected single product of 201 bp in all of them, while in negative samples there was an absence of non-specific amplifications (**Supplementary Figure 1**). In addition, positive and negative results obtained by both conventional methods coincided in all the control samples analyzed. The 35 PCR products sequenced from different bacterial hosts originating from Brazil, Costa Rica, Cuba, and the USA showed sequences identities of 98.5 to 100% with *fusA* gene of ‘*Ca*. Liberibacter’ spp. associated with HLB disease.

### 3.2 Validation of the new real-time RPA

#### 3.2.1 Analytical sensitivity

Analytical sensitivity results, evaluated on healthy crude extracts spiked with known amounts of synthetic dsDNA, showed that the real-time RPA can detect synthetic sequences of the three species associated with HLB (CaLas, CaLam, and CaLaf) with a LOD that was at units of copies per microliter (4 to 8 copies/µl). More consistent LOD results were obtained with synthetic dsDNA of CaLas-gBlocks, in which the three replicates evaluated were positive, than with CaLaf- and CaLam-gBlocks, in which of the three replicates only two and one, respectively, were positive (**Table 1**).

**Table 1:** Analytical sensitivity evaluation of the new real-time RPA protocol, performed with known amounts of synthetic dsDNA spiked in healthy crude extracts of *Citrus sinensis* cv. Lane.

Real- time RPA results (total copy number of target analyzed)*							
Synthetic DNA	Dilutions of healthy extracts of *C. sinensis* cv. Lane spiked with synthetic DNA						
	10^−1^	10^−2^	10^−3^	10^−4^	10^−5^	10^−6^	10^−7^
CaLas-gBlocks	+++	+++	+++	+++	+++	+++	+++
	(4 × 10^5^)	(4 × 10^4^)	(4 × 10^3^)	(4 × 10^2^)	(4 × 10^1^)	(40)	(4)
CaLaf-gBlocks	+++	+++	+++	+++	+++	+++	++-
	(6 × 10^5^)	(6 × 10^4^)	(6 × 10^3^)	(6 × 10^2^)	(6 × 10^1^)	(60)	+- -
CaLam-gBlocks	+++	+++	+++	+++	+++	++-	+–
	(8 × 10^5^)	(8 × 10^4^)	(8 × 10^3^)	(8 × 10^2^)	(8 × 10^1^)	(80)	(8)

RPA, recombinase polymerase amplification.

*Avogadro constant was used to estimate the number of double- stranded DNA (dsDNA) (6.023 × 10^23^ molecules/mol) in each dilution.

Upper row of each box: RPA reaction of each of three replicates (positive reaction, +; negative reaction, -); lower row: copy number as quantified by real-time PCR from de Chaves et al. (2023).

The results of analytical sensitivity on HLB-infected material, diluted in healthy extract, showed amplifications in a time period ranging from 4 to 20 min, with a real-time RPA LOD at tens of copies per microliter (3.95 × 10^1^ to 4.37 × 10^1^ copies/µl), in both crude extracts of citrus plants and insect vectors. With the real-time qPCR by de Chaves et al. (2023), units of copies per microliter (3.95 to 4.37 copies/µl) from total DNA purified were detected (**Table 2**) (**Supplementary Figure 2**). LOD results of real-time RPA in HLB-infected plant material were more consistent than in HLB-infected insect material since in plant material two of three replicates were positive, while in insect material it was only one. With HLB-infected material (non-diluted), similar results were obtained since the new real-time RPA technique was capable of detecting up to tens of copies per microliter, between 2.6 × 10^6^ and 1.08 × 10^1^ copies/µl, but not bacterial titers lower than 7 copies/µl (**Supplementary Table 1**).

**Table 2:** Analytical sensitivity evaluation of the new real-time RPA and absolute quantification by real-time PCR of HLB-infected citrus and insect material 10-fold serial diluted on healthy material.

Host	Dilutions in healthy plant extracts	Real- time RPA	Quantification by real-time PCR (copies/µl)*
		Crude extract	Total DNA purified
*Citrus hystrix* (sample 12112.6)	1:10	+++	2.55 × 10^5^
	1:100	+++	6.67 × 10^4^
	1:1000	+++	4.51 × 10^3^
	1:10,000	+++	4.95 × 10^2^
	1:100,000	++-	2.95 × 10^1^
	1:1.000,000	- - -	3.95
	Healthy plant material	- - -	- - -
*Diaphorina citri* (sample 11661.7)	1:10	+++	6.35 × 10^5^
	1:100	+++	7.62 × 10^4^
	1:1000	+++	2.17 × 10^3^
	1:10,000	+++	3.84 × 10^2^
	1:100,000	+ - -	5.61 × 10^1^
	1:1,000,000	- - -	4.37
	Healthy insect material	- - -	- - -

RPA, recombinase polymerase amplification; HLB, Huanglongbing.

* Absolute quantification performed with efficiency > 95% of real-time PCR by de Chaves et al. (2023).

The results by real-time RPA are coded with + for positive reactions, and - for negative reactions, in three replicates per diltuion of each sample.

#### 3.2.2 Analytical specificity and selectivity

Regarding the inclusivity results of the new real-time RPA, it was able to detect CaLas, CaLaf, and CaLam on HLB-infected samples from nine different origins (São Paulo and Paraná of Brazil, Alajuela of Costa Rica, Florida of the USA, Artemisa of Cuba, and INRAE collection) and five different HLB-hosts (*C. sinensis* cv. Valencia, Rusk citrange, *C. hystrix*, *Citrus* sp., and *D. citri*).

All the sequence analyses of the 35 real-time RPA products, selected as representing different origins and hosts of HLB, corresponded to *fusA* gene of ‘*Ca*. Liberibacter’ species. Identity percentage sequences obtained by BLASTN showed values ranging from 99.2% to 100% with CaLas (isolate A4), 98.5% to 100% with CaLam (isolate São Paulo), and 98.5% to 100% with CaLaf (isolate PTSAPSY). New obtained partial sequences of *fusA* gene were submitted to GenBank database (GenBank accession numbers: OQ507405 to OQ507466).

Concerning the exclusivity, the non-target bacteria *A. vitis* (IVIA 339/26), *A. tumefaciens* (strain C58), *Liberibacter crecens* (strain BT-1), and ‘*Ca*. Liberibacter solanacearum’ did not show amplification with the new real-time RPA protocol. The same results were obtained when evaluating the strains of citrus phytopathogenic bacteria *X. fastidiosa* subsp. *pauca* (strain CFBP 8072), *X. fastidiosa* subsp. *fastidiosa* (IVIA 5770), *X. citri* subsp. *citri* (CFBP 2911), *P. syringae* pv. *syringae* (IVIA 2827), and *S. citri* (NCPPB 3095). Also, there were no specific amplifications in negative samples of *C. sinensis* cv. Lane Late, *C. clementina* cv. Clemenules, *C. limon* cv. Finomesero, *Citrus reshni*, *D. citri*, and *T. erytreae*. These results coincided with the real-time qPCR results obtained (**Supplementary Table 1**).

Regarding the selectivity of the real-time RPA protocol, it was able to detect isolates of ‘*Ca*. Liberibacter’ spp. on five HLB-infected different citrus species and cultivars, as well as different insect specimens of *D. citri*. Specifically, the results showed that the new real-time RPA was able to detect ‘*Ca*. Liberibacter’ spp. in 125 samples of *C. sinensis* cv. Valencia, one Rusk citrange, three *C. hystrix*, three *Citrus* spp., and 35 *D. citri* samples from the USA and Cuba.

#### 3.2.3 Repeatability and reproducibility

Repeatability and reproducibility were assessed by analyzing 10 samples infected with relatively low concentrations of CaLas, with Cqs values ranging from 21.1 to 36.7. Technical replicates of each sample were analyzed using two different fluorimeters, Genie^®^ II and AmplifyRP^®^ XRT, and by two different operators. The Cqs values obtained by real-time qPCR and results obtained by real-time RPA in the two pieces of equipment are shown in **Table 3**. All infected samples showed positive results in both fluorimeters. Only one sample showed different results between the two devices: sample 607 showed negative results in one of the replicates analyzed in Genie^®^ II, while in AmplifyRP^®^ XRT, it was positive in all replicates.

**Table 3:** Evaluation of repeatability and reproducibility of the real-time RPA (in Genie II and AmplifyRP XRT) with 10 samples of Citrus sinensis cv. Valencia plants infected with ‘Candidatus Liberibacter asiaticus’.

Sample ID	Real-time PCR according to de Chaves et al., 2023 (C_q_ average ± SE)	Genie^®^ II	AmplifyRP^®^ XRT
473	23.49 ± 0.24	+++	+++
607	24.17 ± 0.54	++-	+++
614	21.22 ± 0.96	+++	+++
615	22.76 ± 0.24	+++	+++
623	21.76 ± 0.86	+++	+++
627	22.23 ± 0.31	+++	+++
631	21.15 ± 0.45	+++	+++
633	21.91 ± 0.21	+++	+++
635	21.15 ± 0.71	+++	+++
641	22.64 ± 0.23	+++	+++
875	35.87 ± 0.11	++-	++-
874	36.71 ± 0.21	+++	+++
Infected extract of *C. sinensis* cv. Valencia	21.98 ± 0.38	+++	+++
Healthy extract of *C. sinensis* cv. Lane	- - -	- - -	- - -

RPA, recombinase polymerase amplification.

The results by real-time RPA are coded with + for positive reactions, and - for negative reactions, in three replicates per sample.

#### 3.2.4 Diagnostic sensitivity and specificity

The evaluation of diagnostic sensitivity and specificity was carried out by analyzing healthy and HLB-infected samples, both symptomatic and asymptomatic, collected randomly from three different origins (Brazil, Costa Rica, and Spain). A total of 226 citrus samples were analyzed by real-time qPCR (de Chaves et al., 2023) and the new real-time RPA design. Out of the total samples collected, 118 were positive and 88 were negative by real-time PCR, while 99 were positive and 107 were negative by real-time RPA. All partial sequences of *fusA* gene obtained from the 27 real-time RPA positive samples selected corresponded to CaLas and were submitted to the GenBank database (GenBank accession numbers: OQ507440 to OQ507466). Results from a correlation between both detection methods showed that the real-time RPA has a diagnostic sensitivity of 83.89%, a diagnostic specificity of 100%, and a relative accuracy of 91.59% in comparison with the gold standard method (**Table 4**). The agreement between the real-time qPCR (de Chaves et al., 2023) and the real-time RPA developed in this study was almost perfect, as Cohen’s kappa index was 0.83.

**Table 4:** Evaluation of diagnostic sensitivity and specificity of the new real-time RPA by comparison with a validated real-time PCR (de Chaves et al., 2023).

		Real-time PCR according to de Chaves et al. (2023)		
		Positive	Negative	Total
**Real-time RPA**	**Positive**	99	0	99
	**Negative**	19	108	127
	**Total**	118	108	226
**Diagnostic sensitivity**		83.89%		
**Diagnostic specificity**		100%		
**Relative accuracy**		91.59%		

RPA, recombinase polymerase amplification.

## 4 Discussion

The HLB disease and its vectors, aided by intense international trade and climate change, are spreading rapidly, and HLB continues to decimate citrus productivity, with a devastating impact on the crop in important citrus growing areas. In fact, in countries where the disease is present, different control strategies have been carried out without significant success (Andrade et al., 2020; Bassanezi et al., 2020). Therefore, the best strategy against HLB disease remains to prevent the introduction and establishment rather than to cure it (Bové, 2006; Wang, 2020). Therefore, the threat of this disease to free areas, such as the Mediterranean Basin and Australia, requires that preventive measures be taken to at least delay the entry of the bacterium. For this purpose, it is essential to have sensitive, specific, and accurate techniques for early detection and containment. It is also very important that they have potential application at the front line, at the point of need in the field, and that they are rapid and robust with good cost-effectiveness.

The main requirements for a detection technique to be used at the point of care are a specificity of approximately 100% (absence of false positives), a sensitivity as high as possible (limited false- negative results), an easy implementation in a short time, and a limited cost (Cesbron et al., 2022). In this work, a fast on-site detection protocol, based on real-time recombinase polymerase amplification of gene *fusA*, which requires minimal and portable instrumentation and has a short reaction time, has been designed and developed for the detection of the three ‘*Ca.* Liberibacter’ species associated with HLB. Although none of these three species are currently present in Europe, the two main insect vectors are already established and expanding in the Mediterranean Basin (Pérez Otero et al., 2015; Wang, 2020; Ministry of Agriculture and Rural Development of Israel, 2022). This, along with the continued movement of plant material imports worldwide, poses a serious threat to the introduction of HLB-associated bacteria into HLB-free regions. This threat is from both CaLas and CaLaf and, probably to a lesser extent, CaLam. The developed real-time RPA method allows the detection of the three species with excellent sensitivity and specificity, in addition to very fast reaction time, which makes it ideal for inspection in border points, local farms, or greenhouses since its use would increase the possibility of intercepting infected material, both insect and plant tissues.

In recent years, new molecular protocols for HLB detection have been developed and could be conveniently used on-site (Li et al., 2012; Rigano et al., 2014; Ghosh et al., 2016; Wu et al., 2016; Qian et al., 2017; Choi et al., 2018; Stolowicz et al., 2022; Thoraneenitiyan et al., 2022). However, all these protocols have been designed to detect only CaLas and are predominantly based on loop-mediated isothermal DNA amplifications, and only three are based on recombinase polymerase amplification. Although these protocols are highly valuable for on-site detections in areas where CaLas is established, they may not provide complete protection against HLB in regions where the three ‘*Ca*. Liberibacter’ species pose a potential risk. The new real-time RPA method developed in this study can detect CaLas, CaLaf, and CaLam in both plant and insect samples and can be easily applied for on-site detection, as it requires simple, portable equipment and no DNA purification step. All this displays real-time results in as little as 35 min.

The primers and probe designed for this new protocol target *fusA* gene, a highly conserved gene among HLB-associated bacterial species. The sequence is unique, conserved, and highly specific; it matches multiple genomes of each species and has been found to be specific. Moreover, it was observed that primers are specific to target HLB pathogens and do not cross- react with other major citrus pathogens. Unlike the 16S rRNA gene, which is known to produce false- positive results (Morán et al., 2020), the use of *fusA* gene offers a more specific detection method according to the findings of this study. The specificity of primers and probe designed for this new real-time RPA protocol was evaluated both *in silico* and *in vitro*. Results showed a high specificity degree since amplification was obtained with all samples containing HLB-associated ‘*Ca*. Liberibacter’ spp., while no non-specific amplification was obtained in the samples not infected by HLB. The sequencing of the products obtained by conventional PCR showed the selective amplification of CaLas, CaLaf, and CaLam. In addition, the newly developed real-time RPA has been validated following the guidelines set by the EPPO standards (EPPO, 2021a), comparing it with the gold standard method (real-time PCR). This fact is very relevant since it implies that the newly developed real-time RPA is the only protocol for on-site detection of HLB that complies with EPPO standards to date, which is a guarantee of quality management requirements.

The new protocol showed a high analytical sensitivity, both in the evaluations carried out with synthetic dsDNA and in infected plant and insect material. In the case of synthetic dsDNA, the new RPA method was capable of detecting 4–8 copies/μl, while in infected material, it was capable of detecting amounts on the order of 10^1^ copies/μl, which is a value similar to that obtained in other recent works with RPA in other pathosystems (Cesbron et al., 2022). The different sensitivity between synthetic dsDNA and natural samples can be due to the fact that the synthetic dsDNA is not encapsulated in a lipid membrane, contrary to what occurs with naturally infected material, where the DNA target is inside cells. Therefore, it can be said that the reliable detection limit of this protocol is approximately the order of 10^1^ copies/μl, one order of magnitude lower than the LOD of the real-time qPCR with which it was compared (de Chaves et al., 2023). Evaluation of the diagnostic sensitivity of the new RPA method with a random selection of healthy and infected plant samples from three different countries, with a range of different target inoculum levels, revealed a concordance of 83.89% with the gold standard method. The lower sensitivity of the new protocol with respect to real-time qPCR is possibly due to the fact that detection below the order of magnitude of 10^1^ copies/μl is subject in RPA to a random factor, as suggested by the results obtained during the evaluation of the analytical sensitivity.

Regarding the analytical specificity of the new RPA protocol, inclusivity results suggest that it can detect a broad range of HLB-associated ‘*Ca*. Liberibacter’ isolates representing the diversity of the three species, as it was able to recognize target isolates from nine different origins, including São Paolo and Paraná of Brazil, Alajuela of Costa Rica, Florida of the USA, Artemisa of Cuba, and the INRAE collection (France). Furthermore, the new real-time RPA was able to detect ‘*Ca*. Liberibacter’ isolates infecting at least five different plant hosts, such as *C. sinensis* cv. Valencia, Rusk citrange, *C. hystrix*, *Citrus* sp., and *D. citri* insect, suggesting that variations in the host-matrix, assessed in this study, do not affect the throughput of the new protocol. The exclusivity results indicate that the relevant phylogenetically close non-target bacterial species (‘*Ca*. Liberibacter solanacearum’, *L. crescens*, *A. tumefaciens*, and *A. vitis*) and phytopathogenic citrus bacterial species (*X. fastidiosa* subsp. *pauca*, *X. fastidiosa* subsp. *fastidiosa*, *X. citri* subsp. *citri*, and *P. syringae* pv. *syringae*) do not cause the occurrence of non-specific amplifications with the newly developed protocol. Moreover, the diagnostic specificity results showed 100% agreement with the gold standard method.

All these specificity results suggest that the new real-time RPA could detect with high confidence the three HLB-associated bacterial species and different isolates, being able to discriminate from other important citrus pathogens. Finally, regarding the repeatability and reproducibility of the new protocol, the high level of consistency in the results of the technique makes it ideal for obtaining reliable and comparable performance even when the technique is used by different operators and on different fluorimeters. Therefore, all these results suggest that the new real-time RPA protocol could be effectively used as a first rapid screening technique at strategic entry points, mainly in HLB-free regions where the three species of ‘*Ca*. Liberibacter’ is a potential threat.

This new protocol developed in this study is not intended to replace other more sensitive molecular methods, such as real-time PCR, but rather to be an additional tool to support HLB diagnosis within the management systems of this serious disease. It is a prototype with great potential for use at points of care, within HLB prevention strategy plans, and in regions where there is a high risk of introduction or spread of the disease. It can be used as a first screening test for the detection of HLB in suspicious samples, such as symptomatic samples or samples from areas where the disease is present. This prototype overcomes the need for extensive technical expertise and complex infrastructure. In addition, the advantage of not requiring DNA purification provides greater cost-effectiveness than other technologies and allows the end user to feasibly perform screening tests at the point of need.

In conclusion, the results presented in this study demonstrate that the new real-time RPA protocol is a highly sensitive, specific, and accurate tool for the rapid diagnosis of HLB that can be used with easy-to-use portable equipment. This makes it very suitable for rapid point-of-care detection, especially useful for those disease-free countries with a high risk of introduction of any of the three ‘*Ca*. Liberibacter’ species associated with HLB.

## Data availability statement

The datasets presented in this study can be found in online repositories. The names of the repository/repositories and accession number(s) can be found in the article/**Supplementary Material**.

## Author contributions

Conceptualization and methodology: FM and EM-N. Validation: FM, MH-C and SC-R. Formal analysis: FM and EM-N. Investigation: FM, EM-N, MH-C and SC-R. Resources: FM and EM-N. Data curation: FM and EM-N. Writing—original draft preparation: FM. Writing—review and editing: FM, EM-N, MH-C and SC-R. Funding acquisition: EM-N. All authors contributed to the article and approved the submitted version.
