# Supplementary material for: Real-time on-site detection of the three ‘Candidatus Liberibacter’ species associated with HLB disease: a rapid and validated method
Source: Front Plant Sci. 2023 Jun 7;14:1176513. doi: 10.3389/fpls.2023.1176513 (PMC10282772; doi:10.3389/fpls.2023.1176513)
Supplement: Supplementary file 1 [file DataSheet_1.docx]

Supplementary Material

Real-Time On-site Detection of the three `*Candidatus* Liberibacter´ Species associated with HLB disease: A Rapid and Validated Method

Félix Morán^1^, Mario Herrero-Cervera^1^, Sofía Carvajal-Rojas^2^ and Ester Marco-Noales*^1^

^1^Instituto Valenciano de Investigaciones Agrarias (IVIA). Centro de Protección Vegetal y Biotecnología. Unidad de Bacteriología.

^2^Universidad de Costa Rica (UCR). Centro de Investigación en Biología Celular y Molecular (CIBCM). Laboratorio de Fitopatógenos Obligados y sus Vectores (LaFOV).

***Correspondence:**marco_est@gva.es

# Supplementary Data

Sequences of synthetic gBlocks which contain a partial sequence (223 bp) of the *fusA* gene from species `*Candidatus* Liberibacter asiaticus´ (CaLas), `*Candidatus* Liberibacter africanus´ (CaLaf) and `*Candidatus* Liberibacter americanus´ (CaLam) used on analytical sensitivity evaluation of the new real-time RPA developed.

> CaLaf_fusA_RPA

AGAAATCATTTCAACAGAACGATAAAAGTCCGCGCCCATCTTATCCATTTTATTGCAAAATATCACGCGCGGAGTAGAATACTTGTTCGCCTGACGCCATACCGTTTCTGTTTGAGGTTCAACACCAGCATTAGCATCTAACAATGTTATCGCACCATCCGAAACACGAATAGAACGTTCTACTTCCATAGTAAAGTCAACGTGTCCAGGAGTATCAATAATT

> CaLas_fusA_RPA

AGAAATCATCTCAACAGAACGATAAAAATCCGCACCCATCTTATCCATTTTATTGCAAAATATCACACGCGGAACAGAATACTTATCAGCCTGACGCCATACCGTTTCTGTTTGCGGTTCAACACCAGCGTTAGAATCTAATAACGCTATCGCCCCATCCGTAACACGAATAGAACGCTCAACTTCCATAGTAAAATCAACATGCCCAGGAGTATCAATAATT

> CaLam_fusA_RPA

AGAAATCATTTCAACAGATCGATAAAAATCAGCGCCCAGTTTATCCATCTTATTACAAAAGATCATACGAGGAACAGAGTATTTATCCGCCTGACGCCATACAGTCTCTGTTTGAGGCTCAACCCCAGCATTAGAATCCAAAAGAGCAATAGCGCCATCCAAAACCCGAATGGAACGCTCAACTTCCATAGTAAAATCAACGTGACCAGGGGTATCAATAATA

# Supplementary Figures and Tables

## Supplementary Tables

**Table S1.** Analytical specificity, selectivity and limit of detection results in samples naturally infected analysed by real time PCR (de Chaves et al., 2023) and real-time RPA

| **Bacterial specie identified^1^** | **Sample ID or Strain ID** | **Host** | **Country** | **Region** | **Quantification by**  **real-time PCR^2^** | **Real-time RPA** |
| --- | --- | --- | --- | --- | --- | --- |
| CaLas | 4119 | *Citrus sinensis*  cv. Valencia | Brazil | San Paulo | 2.39 x 10^3^ | +++ |
|  | 4123 |  |  |  | 5.38 x 10^3^ | +++**^3^** |
|  | 4127 |  |  |  | 4.69 x 10^4^ | +++**^3^** |
|  | 4217 |  |  |  | 6.11 x 10^2^ | +++**^3^** |
|  | 4224 |  |  |  | 5.52 x 10^3^ | +++**^3^** |
|  | 4226 |  |  |  | 3.49 x 10^4^ | +++ |
|  | 4227 |  |  |  | 4.37 x 10^4^ | ++- |
|  | 4233 |  |  |  | 6.86 x 10^2^ | +++ |
|  | 4257 |  |  |  | 6.12 x 10^4^ | +++**^3^** |
|  | 4258 |  |  |  | 7.71 x 10^4^ | +++**^3^** |
|  | 4259 |  |  |  | 6.12 x 10^4^ | +++ |
| CaLam | Lam 31 |  |  |  | 5.13 x 10^2^ | +++**^3^** |
|  | Lam 33 |  |  |  | 3.53 x 10^3^ | +++**^3^** |
| CaLas | 4324 |  |  | Paraná | 7.30 | --- |
|  | 4327 |  |  |  | 1.38 x 10^3^ | +++**^3^** |
|  | 4425 |  |  |  | 4.31 | --- |
|  | 4432 |  |  |  | 7.12 x 10^1^ | ++- |
|  | 4447 |  |  |  | 4.56 x 10^1^ | ++- |
|  | 4448 |  |  |  | 6.12 x 10^1^ | ++- |
|  | 4449 |  |  |  | 6.32 | --- |
|  | 4450 |  |  |  | 4.46 x 10^1^ | ++- |
|  | 4452 |  |  |  | 1.82 x 10^1^ | +++ |
|  | 4453 |  |  |  | 2.23 | +-- |
|  | 4456 |  |  |  | 4.90 x 10^1^ | ++- |
|  | 4528 |  |  |  | 5.50 | --- |
|  | 4535 |  |  |  | 5.52 x 10^3^ | +++**^3^** |
|  | 4537 |  |  |  | 1.22 x 10^4^ | +++**^3^** |
|  | 123 | *Citrus sinensis*  cv. Valencia | Costa Rica | Alajuela | 7.67 x 10^2^ | +++**^3^** |
|  | 124 |  |  |  | 2.31 x 10^3^ | +++**^3^** |
|  | 141 |  |  |  | 1.78 x 10^4^ | +++**^3^** |
| No-CaLspp | Cs-1 | *Citrus sinensis*  cv. Lane late | Spain | Valencia | - | --- |
|  | Cs-2 |  |  |  | - | --- |
|  | Cs-3 |  |  |  | - | --- |
|  | Cs-4 | *Citrus clementina*  cv. Clemenules |  |  | - | --- |
|  | Cs-5 |  |  |  | - | --- |
|  | Cc-1 |  |  |  | - | --- |
|  | Cc-2 |  |  |  | - | --- |
|  | Cc-3 |  |  |  | - | --- |
|  | Cl-1 | *Citrus limon*  cv. Fino mesero |  |  | - | --- |
|  | 11661-3 | *Diaphorina citri* | US | Florida | - | --- |
| CaLas | 11661-2 |  |  |  | 4.61 x 10^1^ | ++- |
|  | 11661-1 |  |  |  | 2.73 x 10^1^ | ++- |
|  | 11661-4 |  |  |  | 1.17 x 10^2^ | +++ |
|  | 11661-5 |  |  |  | 4.70 x 10^2^ | +++ |
|  | 11661-6 |  |  |  | 7.64 | +-- |
|  | 11661-7 |  |  |  | 4,37 x 10^6^ | +++**^3^** |
|  | 11661-14 |  |  |  | 6.86 x 10^4^ | +++ |
|  | 11661-17 |  |  |  | 2.59 x 10^4^ | +++ |
|  | 11661-26 |  |  |  | 7.74 x 10^2^ | +++ |
|  | 11662-1 |  |  |  | 6.23 x 10^4^ | +++**^3^** |
|  | 11662-2 |  |  |  | 5.72 x 10^1^ | ++- |
|  | 11662-3 |  |  |  | 5.04 x 10^1^ | +++ |
|  | 11662-5 |  |  |  | 4.65 x 10^4^ | +++**^3^** |
|  | 11662-4 |  |  |  | 4.60 x 10^1^ | ++- |
|  | 11662-6 |  |  |  | 1.43 x 10^4^ | +++**^3^** |
|  | 11662-7 |  |  |  | 1.16 x 10^4^ | +++**^3^** |
|  | 11662-8 |  |  |  | 4.67 x 10^1^ | ++- |
|  | 11662-9 |  |  |  | 3.77 x 10^5^ | +++**^3^** |
|  | 11662-10 |  |  |  | 9.04 x 10^1^ | +++ |
|  | 11662-11 |  |  |  | 2.08 x 10^1^ | ++- |
|  | 11662-13 |  |  |  | 1.73 x 10^2^ | +++ |
|  | 11662-14 |  |  |  | 3.76 x 10^3^ | +++**^3^** |
|  | 11662-15 |  |  |  | 3.39 x 10^2^ | +++ |
|  | 11662-17 |  |  |  | 1.86 x 10^4^ | +++**^3^** |
|  | 11662-18 |  |  |  | 4.59 x 10^1^ | ++- |
|  | 11662-19 |  |  |  | 8.68 x 10^1^ | ++- |
|  | 11662-20 |  |  |  | 2.80 x 10^4^ | +++**^3^** |
|  | 11662-21 |  |  |  | 5.75 x 10^5^ | +++**^3^** |
| No-CaLspp | 11533-1 | *Trioza erytreae* | South Africa |  | - | ---- |
|  | 11533-2 |  |  |  | - | --- |
|  | 11533-3 |  |  |  | - | --- |
|  | 11533-4 |  |  |  | - | --- |
|  | 11533-5 |  |  |  | - | --- |
|  | 11533-6 |  |  |  | - | --- |
|  | 11533-7 |  |  |  | - | --- |
|  | 11533-8 |  |  |  | - | --- |
|  | 11533-9 |  |  |  | - | --- |
|  | 11533-10 |  |  |  | - | --- |
| CaLas | 12112.2 | Rusk citrange | Cuba | Artemisa | 6.19 | --- |
|  | 12112.1 |  |  |  | 1.13 x 10^1^ | +--**^3^** |
| No-CaLspp | 12112.3 | *Citrus reshni* |  |  | - | --- |
| CaLas | 12112.4 | *Citrus hystrix* |  |  | 8.37 x 10^4^ | +++ |
|  | 12112.5 |  |  |  | 1.62 x 10^5^ | +++**^3^** |
|  | 12112.6 |  |  |  | 2.6 x10^6^ | +++ |
| No-CaLspp | 12112.7 | *Diaphorina citri* |  |  | - | --- |
|  | 12112.12 |  |  |  | - | --- |
|  | 12112.14 |  |  |  | - | --- |
|  | 12112.19 |  |  |  | - | --- |
|  | 12112.21 |  |  |  | - | --- |
| CaLas | 12112.8 |  |  |  | 4.77 x 10^5^ | +++**^3^** |
|  | 12112.13 |  |  |  | 5.41 | --- |
|  | 12112.9 |  |  |  | 7.23 x10^1^ | ++-**^3^** |
|  | 12112.15 |  |  |  | 7.99 x 10^1^ | +--**^3^** |
|  | 12112.16 |  |  |  | 4.31 | +--**^3^** |
|  | 12112.17 |  |  |  | 1.37 x 10^1^ | ++-**^3^** |
|  | 12112.18 |  |  |  | 3.48 | --- |
|  | 12112.10 |  |  |  | 2.45 x 10^1^ | +++**^3^** |
|  | 12112.20 |  |  |  | 3.58 | --- |
|  | 12112.11 |  |  |  | 1.08 x 10^1^ | ++- |
| CaLaf | Laf-10 | *Citrus* spp. | INRAE collection | | 5.57 x 10^3^ | +++**^3^** |
|  | Laf-2 |  |  |  | 3,34 x 10^4^ | +++**^3^** |
|  | Laf-3 |  |  |  | 2.78 x 10^3^ | +++**^3^** |
| No-CaLspp | 12528-1 | *Trioza erytreae* | Spain | Vigo | - | --- |
|  | 12528-2 |  |  |  | - | --- |
|  | 12528-3 |  |  |  | - | --- |
|  | 12528-4 |  |  |  | - | --- |
|  | 12528-5 |  |  |  | - | --- |
|  | 12528-6 |  |  |  | - | --- |
|  | 12528-7 |  |  |  | - | --- |
|  | 12528-8 |  |  |  | - | --- |
|  | 12528-9 |  |  |  | - | --- |
|  | 12528-10 |  |  |  | - | --- |
|  | 12528-11 |  |  |  | - | --- |
|  | 12528-12 |  |  |  | - | --- |
|  | 12528-13 |  |  |  | - | --- |
|  | 12528-14 |  |  |  | - | --- |
|  | 12528-15 |  |  |  | - | --- |
|  | 12528-16 |  |  |  | - | --- |
|  | 12528-17 |  |  |  | - | --- |
|  | 12528-18 |  |  |  | - | --- |
|  | 12528-19 |  |  |  | - | --- |
|  | 12528-20 |  |  |  | - | --- |
|  | 12528-21 |  |  |  | - | --- |
|  | 12528-22 |  |  |  | - | --- |
|  | 12528-23 |  |  |  | - | --- |
|  | 12528-24 |  |  |  | - | --- |
|  | 12528-25 |  |  |  | - | --- |
|  | 12528-26 |  |  |  | - | --- |
|  | 12528-27 |  |  |  | - | --- |
|  | 12528-28 |  |  |  | - | --- |
|  | 12528-29 |  |  |  | - | --- |
| `*Ca*. Liberibacter solanacearum´ | 579/22 | *Solanum tuberosum* |  | Salamanca | 1.98 x 10^6^ | --- |
|  | CaLsol-Carrot | *Daucus carota* |  | Valencia | 3.45 x 10^6^ | --- |
| *Liberibacter crescens* | BT-1 | *Carica pentagona* | Puerto Rico | - | - | --- |
| *Agrobacterium tumefaciens* | C58/ ATCC 33970 | *Prunus avium* | USA | New York | - | --- |
| *Agrobacterium vitis* | IVIA 339.26 | *Vitis vinifera* | Spain | Orense | - | --- |
| *Xylella fastidiosa subsp. pauca* | CFBP 8072 | *Coffea arabica* | Ecuador | Unknow | - | --- |
| *Xylella fastidiosa subsp. fastidiosa* | IVIA 5770 | *Vitis vinifera* | Spain | Mallorca | - | --- |
| *Xanthomonas citri subsp. citri* | CFBP 2911 | *Citrus* spp. | Pakistan | Unknow | - | --- |
| *Spiroplasma citri* | NCPPB 3095 | *Citrus* spp. | Unknow | Unknow | - | --- |
| *Pseudomonas syringae* pv.*syringae* | IVIA 2827 | *Citrus reticulata* | Spain | Huelva | - | --- |

**^1^** `*Candidatus* Liberibacter´ specie identification by 16S rRNA gene sequencing according to Moran et al., (2020)

**^2^** Absolute quantitation by real-time PCR according to de Chaves et al., (2023) with an efficiency > 95%

**^3^** Control samples selected for *in vitro* specificity evaluation by partial sequencing of the *fusA* gene

## Supplementary Figures

**Supplementary Figure 1.** Agarose gel electrophoresis of conventional PCR products amplified with RPA primers (RPA-HLB-F2/ RPA-HLB-R2), of 35 control HLB- infected samples from citrus and in insect vectors. Lane and sample ID. **1:** 4123, **2:** 4127, **3:** 4217, **4:** 4224, **5:** 4257, **6:** 4258, **7:** Lam31, **8:** Lam33, **9:** 4327, **10:** 4535, **11:** 4537, **12:** 123, **13:** 124, **14:** 141, **15:** 11661-7, **16:** 11662-1, **17:** 11662-5, **18:** 11662-6, **19:** 11662-7, **20:** 11662-9, **21:** 11662-14, **22:** 11662-17, **23:** 11662-20, **24:** 11662-21, **25:** 12112.1, **26:** 12112.5, **27:** 12112.8, **28:** 12112.9, **29:** 12112.15, **30:** 12112.16, **31:** 12112.17, **32:** 12112.10, **33:** Laf-10, **34:** Laf-2, **35:** Laf-3, **36:** Positive amplification control (PAC), **37:** *Liberibacter crescens* (BT-1), **38:** *Agrobacterium tumefaciens* (C58), **39:** Positive isolation control (PIC), **40:** PAC, **41:** PAC, **42:** PAC, **43:** PAC, **44:** *Agrobacterium vitis* (IVIA 339.26), **45:** *Xylella fastidiosa* subsp. *pauca* (CFBP 8072), **46:** *Spiroplasma citri* (NCPPB 3095)*,* **47:** *Xanthomonas citri* subsp*. citri* (CFBP 2911) **48:** *Pseudomonas syringae* pv. *syringae* (IVIA 2827).

**A.**

**B.**

**Supplementary Figure 2.** Analytical sensitivity of the new real-time RPA protocol with ten-fold serial diluted extracts of HLB-infected samples from citrus (A) or insect (B). Dilutions were performed on healthy citrus or insect material, respectively.
